# Case report: Lymph node metastases of breast cancer and thyroid cancer encountered in axilla

**DOI:** 10.3389/fonc.2022.983996

**Published:** 2022-09-30

**Authors:** Rihan Li, Qingfu Zhang, Dongdong Feng, Feng Jin, Siyuan Han, Xinmiao Yu

**Affiliations:** ^1^ Department of Breast Surgery, The First Hospital of China Medical University, Shenyang, China; ^2^ Department of Breast and Reconstructive Surgery, The First Hospital of China Medical University, Shenyang, China; ^3^ Department of Pathology, The First Hospital of China Medical University, Shenyang, China

**Keywords:** breast cancer, thyroid cancer, axillary lymph node metastasis, synchronous cancer, case report

## Abstract

Occurrences of breast cancer and thyroid cancer metachronously or synchronously are common for women, but axillary lymph node metastasis from both cancers is rarely seen. We report a patient who had two metastatic lymph nodes from papillary thyroid carcinoma after axillary lymph node dissection with mastectomy. Papillary thyroid carcinoma diagnosis was ensured after thyroidectomy. A literature review revealed that even the co-occurrence of breast cancer and thyroid cancer is not rare, but the etiology behind this phenomenon is not elucidated well. Genetic disorders, thyroid dysfunction, and hormone receptors may be relevant. Considering the rareness of axillary lymph node metastasis of thyroid cancer, adjuvant therapy and surgery treatment for this kind of case should be considered elaborately.

## Introduction

Breast cancer (BC) has become the most prevalent cancer for women, whereas thyroid cancer (TC) incidence rates continue to be high (1), with some common risk factors like genetic and hereditary factors, estrogen effects, and unhealthy modern lifestyles (2, 3). There were 281,550 (30% of total female cancer) new cases of breast cancer and 32,130 (3% of total female cancer) new cases of thyroid cancer diagnosed in the United States in 2021 (1). The number of new cases of BC and TC in Chinese women is 429,105 (19.5%) and 169,771 (7.7%), respectively, according to data updated for 2022 (4). To our relief, the data in China from 2003 to 2015 demonstrated that the cancer survival of those with BC and TC keep increasing, which is 2.5% and 5.4%, respectively (5).

As cancer research and medical treatment have advanced, the incidence of multiple primary tumors is rising due to early diagnosis and improved survival (6). Occurrences of breast and thyroid cancer metachronously or synchronously are one of the most common multiple primary tumors. It has been documented for many years. In 1966, L. J. Chalstrey and B. Benjamin reported the finding of a higher incidence of BC in TC patients (7). A weak correlation was discovered between the incidence rates of breast and thyroid cancer by L. Eric et al. A linear trend line showed that for every 10 new cases of breast cancer, there were 1.6 new cases of thyroid cancer for every 100,000 women (8). Patients with a history of breast cancer are more likely to be diagnosed with aggressive follicular thyroid cancer than with papillary thyroid cancer (9). Notably, male breast cancer patients are more likely to develop thyroid cancer than female patients, and vice versa (10). The risk factors of occurrences of BC and TC metachronously or synchronously have been first described in 1987 (11), but the possible linkage between them is still unelucidated (8, 12). The etiology and risk factors also have been investigated without certain conclusions.

Lymph node metastases (LNMs) are common in BC and TC, which dictates the prognosis. However, LNMs happening in the same site from both cancers are rarely seen. In this article, we present an extremely rare case of synchronous breast and thyroid cancers with axillary metastatic lymph nodes from both cancers. Meanwhile, it is the first case ever reported with thyroid cancer lymph node skip metastasis to the opposite side even without cervical metastasis of both cancers.

## Case presentation

A 31-year-old woman found a palpable lump in her right breast for a week without a history of trauma or surgery. The patient was diagnosed with hyperthyroidism 10 years ago. The Doppler ultrasound test of the thyroid gland and neck revealed no nodules. She was treated regularly with methimazole. Four years ago, she discontinued the medication after the follow-up examination suggested normal thyroid hormone function. Until she came to our hospital this time, she had not taken any thyroid-related tests or examinations in the last 4 years. No family member of hers ever suffered from cancer before.

**Figure 1:**
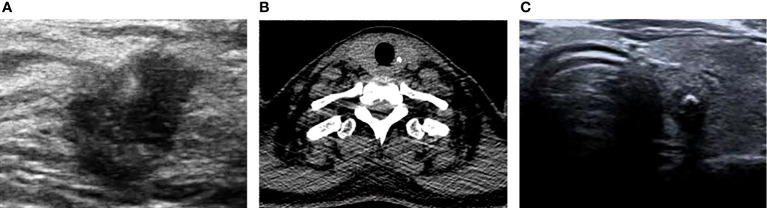
The Doppler ultrasound test of the breast and axilla demonstrated a large, 1.85 × 1.12 × 1.54 cm   retro-areolar lump at the 8 o’clock position **(A)**. The CT scan of the thyroid presented a 4-mm calcium lesion in the left lobular **(B)**. The Doppler ultrasound test of the thyroid gland showed a large, 0.65 × 0.58 × 0.65 cm TI-RADS 4a solid nodule with calcification found in the middle-upper part of the left lobular **(C)**.

Physical examination revealed a large, nearly 2-cm lump at the inferolateral region under the right areolar with a firm and movable character. No tenderness or nipple discharge was found. There is no palpable lymph node in the axilla or other related regions. The Doppler ultrasound test of the breast and axilla demonstrated a large, 1.85 × 1.12 × 1.54 cm   retro-areolar lump at the 8 o’clock position. No swollen or suspicious lymph node was found in axilla drainage areas both laterally. The lump was considered an intraductal lesion or an occupational tumor-leveled BI-RADS 4b (**Figure 1**). Because of economic reasons, the patient refused PET/CT test.

The pathological result confirmed an invasive breast cancer after a tumor resection biopsy. As the patient did not want any oncoplastic surgery or breast reconstruction surgery, right breast resection and sentinel lymph node biopsy were chosen to be performed. During the surgery, two sentinel lymph nodes were found with breast cancer metastasis. Therefore, right axillary lymph node clearance was performed consequently.

**Figure 2:**
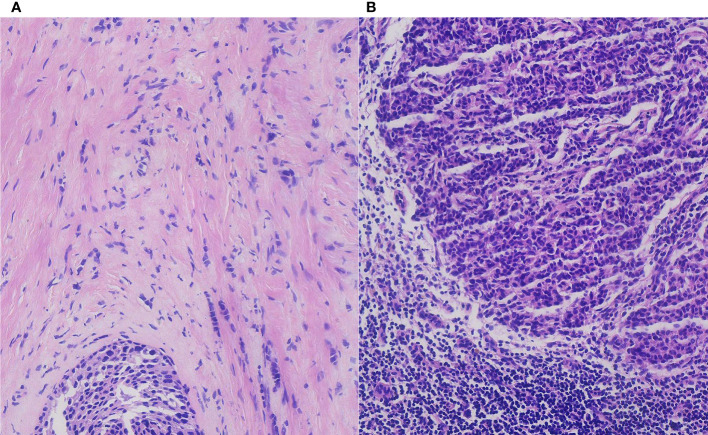
The pathological result demonstrated an invasive breast carcinoma with ductal carcinoma *in situ* **(A)** and breast cancer axillary lymph node macro-metastasis in H&E staining **(B)** (H&E, ×200 original magnification).

The final pathological results demonstrated a large, 12-mm non-special type invasive breast carcinoma (grade 2), with about 20% ductal carcinoma *in situ* (**Figure 2**). Immunohistochemically, a luminal-type breast cancer that is estrogen receptor positive (70%), progesterone receptor positive (90%), C-erB-2 negative (−), and Ki-67 index (15%) was shown.

**Figure 3:**
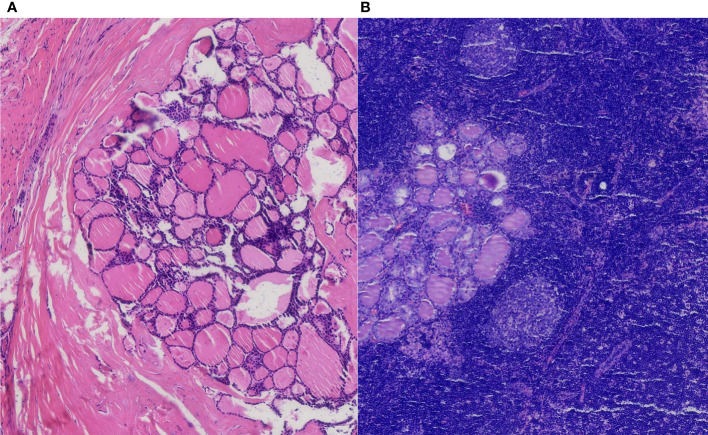
The pathological result of the thyroid papillary carcinoma nodule with an intact membrane **(A)**. Thyroid cancer axillary lymph node metastasis in H&E staining **(B)** (H&E, ×100 original magnification).

Regarding the lymph nodes in the axilla area, 14 lymph nodes were found eventually. The pathological examination found no more breast cancer metastasis with the exception of the two breast cancer macro-metastasis sentinel lymph nodes. To our surprise, two of these lymph nodes presented metastasis from thyroid papillary cancer unexpectedly. Microscopically, breast cancer cells in the lymph nodes were arranged in a solid nest-like or sheet-like pattern typically, with disordered cell polarity, deep-stained nuclei, eosinophilic cytoplasm, and visible nuclear fission images (**Figure 2**). Regarding the thyroid cancer metastatic lymph nodes, the distribution of tumor cells in the lymph nodes presented a follicular pattern, and thyroid glia were seen in the follicles apparently. The nuclei were enlarged and transparent with a hairy glass-like appearance. The nuclear outline was irregular, and nuclear groove formation and pseudo-inclusion bodies were seen (**Figure 3**).

To further investigate the origin of thyroid cancer metastatic lymph nodes in the axilla, a Doppler ultrasound test of the thyroid gland and neck was performed to discover thyroid lesions. A large, 0.65 × 0.58 × 0.65 cm TI-RADS 4a solid nodule with calcification was found in the middle-upper part of the left lobular of the thyroid gland, which is the opposite side of lymph node metastasis (**Figure 1**). The CT scan of the thyroid presented a 4-mm calcium lesion in the left lobular (**Figure 1**). The thyroglobulin (TG) is 2.31 ng/ml. Other biochemical tests showed a normal level of free triiodothyronine (FT3) and free thyroxine (FT4) and an increase in thyroid peroxidase antibody (TPOAB) and thyroglobulin antibodies (TGAB) (**Table 1**).

**Table 1:** The biochemical test results of the patient with thyroid cancer before and after surgery (20 Jan.).

	18 Jan.	14 Feb.	14 Mar.	18 Apr.	11 Jul.
FT3 (pmol/L)	4.58	4.51	4.36	5.98	5.81
FT4 (pmol/L)	15.37	16.13	15.74	22.32	19.69
TSH (uIU/ml)	0.3084	1.5188	1.8966	0.0311	0.0563
TPO (IU/ml)	9.67	–	–	–	–
TGAB (IU/ml)	–	26.92	–	–	2.66
TG (IU/ml)	13.59	<0.04	–	–	<0.04

TG level has an obvious decline after surgery. The FT3 and FT4 were controlled well with the administration of Euthyrox. Considering the high recurrence rate of the patient, the TSH level decreased to under 0.1 uIU/ml in recent follow-ups.-, untested; FT3, free triiodothyronine; FT4, free thyroxine; TSH, thyroid-stimulating hormone; TPO, thyroid peroxidase; TGAB, thyroglobulin antibodies; TG, thyroglobulin.

Considering skip metastasis to the opposite axillary lymph nodes, the patient underwent a bilateral thyroidectomy and bilateral central area lymph node clearance (cervical region VI). The pathological result of thyroid cancer confirmed papillary carcinoma (BRAF positive) (**Figure 3**). The gray-white rigid nodular in the left thyroid lobular is 0.6 cm without capsule invasion. No vascular cancer thrombus or nerve invasion was seen, and no cancerous tissue was seen in the right lobe of the thyroid. No metastatic lymph nodes were seen in the left neck, anterior laryngeal tissue, or right neck.

After surgery, six cycles of standard chemotherapy with paclitaxel and cyclophosphamide were performed according to the treatment guideline of the Chinese Anti-Cancer Association, Committee of Breast Cancer Society (13). The patient also received antiestrogen therapy with ovarian function suppression and tamoxifen for long-term control of breast cancer. Radiotherapy was administrated to decrease the risk of axillary metastasis. Regarding thyroid cancer, a lifetime Euthyrox medication was needed, and radioactive iodine treatment was performed after a comprehensive evaluation.

**Figure 4:**
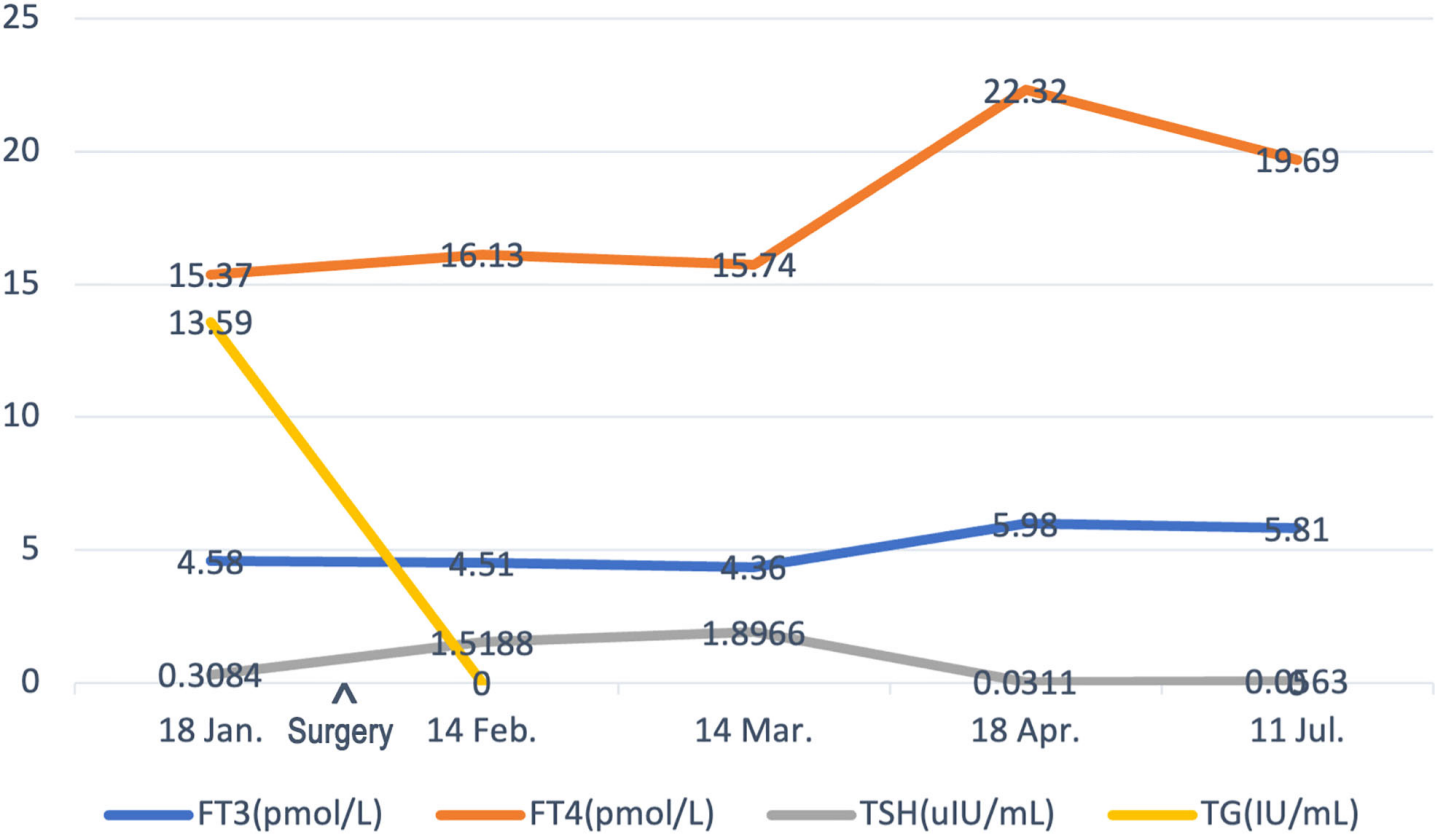
The timeline showcases the results of FT3, FT4, TSH, and TG levels of the patient before surgery and subsequent follow-ups. FT3, free triiodothyronine; FT4, free thyroxine; TSH, thyroid-stimulating hormone; TG, thyroglobulin.

With a follow-up of 8 months, no sign of BC or TC recurrence and metastasis has been found. The ultrasound examination of the breast, thyroid, and related lymph node regions presented no positive signs. TG and TGAB showed an obvious decrease after surgery (**Table 1**). As the patient had a high recurrence rate, the thyroid-stimulating hormone (TSH) level is controlled well according to the 2015 ATA guidelines (14) (**Figure 4**). To date, no postoperative complications such as lymphedema, hoarseness, or hypocalcemia have been identified.

## Discussion

To our knowledge, this is the first case including three characteristics. Firstly, BC and TC occurred synchronously. Secondly, axillary lymph nodes were metastasized from BC and TC concurrently. Lastly, TC skip metastasized to the opposite side axilla without cervical lymph node metastasis. As a case has never been reported before, its potential mechanisms of it have not yet been thoroughly explored. However, we can analyze the underlying correlations and pathogenesis from the following components.

### Breast cancer and thyroid cancer synchronous occurrence

Synchronous multiple primary cancers are defined as the second primary cancers that occurred within 2 months of the first primary tumor (15, 16); survivors of breast cancer had the highest risk of developing synchronous multiple primary cancers (16). In the aspect of BC and TC synchronous occurrence, most scholars accept the time interval of BC and TC as within 6 months (17, 18). In a study in China involving 18,732 first-time BC patients and 12,877 female TC patients, it was observed that synchronous thyroid cancer involved 0.28% of BC patients and synchronous breast cancer involved 0.4% of TC patients (19).

The location of breast cancer including the upper-outer quarter, central portion, and overlapping lesions demonstrated a significant increase in TC development. Higher-grade breast cancer (≥grade 2) also increased the risk to develop TC significantly. Considering hormone receptors, there is a higher risk of luminal-type breast cancer with positive estrogen receptor (ER) and progesterone receptor (PR) (17). Our case was in accordance with the risk factors mentioned before with a grade 2 retro areolar invasive carcinoma and ER and PR positive.

Regarding the potential mechanisms behind BC and TC synchronous occurrence, some genetic factors may shed light on the links between them. First-degree relatives of breast cancer patients are more likely to acquire thyroid cancer, according to a Swedish study of patients (20). Breast and thyroid cancer risks are both increased by a germline PTEN gene abnormality in Cowden’s syndrome, which also causes endometrial and gastrointestinal cancers (21).. Phosphatase and tensin homolog encoded by *PTEN* inhibits the catalytic activity of the enzyme PI3K. The activation of the PI3K–AKT signal pathway promotes survival, proliferation, and migration to drive tumorigenesis (22). Ikeda et al. identified PARP4 as a possible susceptibility gene for primary thyroid and breast cancer (23). Using next-generation sequencing of 112 hereditary cancer risk genes, B. Bakos et al. studied the genetic profiles of patients with BC and TC. The results showed that synchronous BC and TC may result from a genetic predisposition and are likely associated with the burden of carcinogenic single-nucleotide polymorphisms (SNPs) rather than a single gene mutation (24).

In our case, the patient had a hyperthyroidism history of 10 years. It is suspicious that BC and TC synchronous occurrence is associated with thyroid disease or disruptions of thyroid gland functions. Some observational studies with different scales of samples indicate an association between thyroid dysfunction and breast cancer risk. Women with hyperthyroidism had a higher risk of developing breast cancer, while those with hypothyroidism had a reduced risk (25–28). However, these outcomes failed to reach a consensus with other studies (29, 30). Whether the severity of hyperthyroidism or hypothyroidism, plans and length of treatment, or other etiological agents contribute to these results needs further studies.

Due to the crucial roles that the thyroid hormone receptor (TR) and the ER both play in TC and BC (31–33), there might be an association between the co-occurrence of BC and TC with TR and ER. The thyroid hormone receptor β (TRβ) exists in both breast and thyroid glands and is also a tumor suppressor (34). Studies found that TRβ suppresses breast tumorigenesis (35, 36). TRβ inhibits the self-renewal capacity of breast cancer stem cells (37). Oncogene *RUNX2* whose expression is inhibited by TRβ is shared by TC and BC (36). Regarding ER, H. Y. Ahn et al. found that most patients with PTC in Korea were found to exhibit positive expression for ER or PR (38). According to a study by Y. A. Kim et al., the BC and TC group showed higher levels of TR expression and were ER positive than the BC control group (39)..

### Axilla metastasis of breast cancer and thyroid cancer

Axillary lymph node metastasis (ALNM) along the lymph fluid drainage pathway of breast tissue is common in breast cancer patients (40). The most crucial indicator of overall survival and recurrence is the number and location of positive lymph nodes (LNs), which also direct therapeutic choices (41). Regarding lymph node metastasis in thyroid cancer, cancer cells will metastasize to the central region first, followed by the lateral cervical region. It is also an important indicator of prognosis and surgery suggestions (42). In our case, the metastatic lymph nodes from thyroid cancer should be defined as skip metastasis. Skip metastasis is the term used to describe the unusual phenomenon of lateral lymph node metastases without the central compartment of the neck in thyroid cancer. In papillary thyroid carcinoma (PTC), which is the most common thyroid malignancy, this type of metastasis is not rare (43, 44). Research of 450 PTC patients found that skip metastasis was substantially correlated with the upper location of the primary tumor, a primary tumor size ≤10 mm, and capsule invasion (43). Female sex is also an independent risk factor for skip metastases (45).

Axillary lymph node metastases in thyroid cancer are uncommon since the neck and axilla lymphatic systems hardly ever communicate with one another directly. Unlike the skip metastasis in our case, most of the cases with ALNM had cervical LN metastases or distant metastases (46, 47), which means the cancer cells may metastasize forward following the lymphatic drainage step by step. Only 20 cases of well- or poorly differentiated thyroid cancer have been reported about ALNM to date, with papillary carcinoma being found in 16 of those cases, according to A. Suehiro et al. (47). Unlike no lymph node invasion in the neck of our case, the seven patients they treated all had neck lymph node metastasis. A recent report of metastatic thyroid cancer diagnosed during breast cancer axillary sentinel node biopsy also demonstrated an invasive follicular variant of papillary thyroid cancer with lymphovascular invasion (48). Notably, in our case, the thyroid cancer lesion appeared on the left side with a minor size without any cervical lymph node metastasis, and there is no lymphovascular or neuroinvasion appearance. However, skip metastasis of the lymph node was found in the right axilla opposite to the primary tumor. Meanwhile, these thyroid cancer metastatic lymph nodes encountered the lymph nodes that metastasized from a T1 breast cancer nodule. Through our data retrieval, we found that this kind of presentation has never been documented before. The potential mechanisms behind this situation still need to be discovered by further study.

### Treatment of breast cancer and thyroid cancer co-occurrence

Noting that co-occurrence of BC and TC patients is not uncommon, there is a necessity to preclude thyroid malignancy after breast cancer is diagnosed and vice versa. Fine-needle cytology could be included during diagnosis to identify certain pathological results (49, 50). Surgery resection is necessary for patients with TC and BC. Mastectomy surgery and ALN clearance for breast cancer, as total thyroidectomy and selective neck dissection, are recommended (47). In the aspects of adjuvant therapy, chemotherapy, targeted therapy, radiotherapy, and endocrine therapy are needed to correspond to the breast cancer pathological results of patients (51). Radioactive iodine (^131^I) therapy should be considered according to either the TNM stage of TC or serum TG level and ^131^I whole-body scan results for reducing recurrence risk. A lifetime Euthyrox administration is needed for patients who undergo bilateral thyroidectomy. The TSH level of these patients should be controlled under 0.1 mU/L due to the high rate of recurrence (14). Molecularly targeted drugs may also be a complement to surgical treatment (14). As these cases are extremely rare, more cases like ours need to be published. The treatment methods and suggestions may be advanced through larger populations and longer observations.

## Conclusions

Only a few cases of axillary lymph node metastases from BC and TC synchronous occurrence have been reported. However, the appearance of metachronous or synchronous breast and thyroid cancers is common, and the mechanisms of the relationship behind them are still unelucidated. It is worth noting that there is a possibility of misdiagnosis of synchronous cancer when BC or TC is diagnosed. Further studies are needed to reveal the mechanisms of synchronous cancer and metastases in order to make better therapeutic choices.

## Data availability statement

The raw data presented in the study will be made available by the authors without undue reservation.

## Ethics statement

Written informed consent was obtained from the patient for the publication of any potentially identifiable images or data included in this article.

## Author contributions

LR and YX conceived and designed the study. ZQ, YX, FD, and LR diagnosed and treated the patient. LR wrote the original draft of the manuscript. YX, ZQ, HS, and JF revised the manuscript. All authors contributed to the article and approved the submitted version.

## Funding

This work was supported by Wu Jieping Medical Foundation (320.6750.2021-10-108).

## Acknowledgments

We thank the patient and doctors for participating in this study. We thank all the clinicians and nurses involved in the diagnosis, treatment, and further follow-up.

## Conflict of interest

The authors declare that the research was conducted in the absence of any commercial or financial relationships that could be construed as a potential conflict of interest.

## Publisher’s note

All claims expressed in this article are solely those of the authors and do not necessarily represent those of their affiliated organizations, or those of the publisher, the editors and the reviewers. Any product that may be evaluated in this article, or claim that may be made by its manufacturer, is not guaranteed or endorsed by the publisher.
